# Dietary supplementation with selenium yeast during early pregnancy enhances litter size in association with antioxidant capacity, progesterone synthesis, and gut microbiota in sows

**DOI:** 10.1128/spectrum.01303-26

**Published:** 2026-07-13

**Authors:** Yan Chen, Nan Chen, Zhenhong Yan, Yunbo Yang, Lingling Sun, Rui Chen, Yifan Hu, Haoyi Jiang, Xianghua Yan

**Affiliations:** 1 National Key Laboratory of Agricultural Microbiology, Frontiers Science Center for Animal Breeding and Sustainable Production, Hubei Hongshan Laboratory, College of Animal Sciences and Technology, Huazhong Agricultural University 627716, Wuhan, Hubei, China; 2 National Engineering Research Center for Green Feed and Healthy Breeding, Key Laboratory of Animal Molecular Nutrition, Ministry of Education, Key Laboratory of Animal Nutrition and Feed Science (Eastern of China), Ministry of Agriculture and Rural Affairs, Zhejiang Key Laboratory of Nutrition and Breeding for High-quality Animal Products Institute of Feed Science, College of Animal Sciences, Zhejiang University 366125, Hangzhou, Zhejiang, China; University of Arkansas Fayetteville, Fayetteville, Arkansas, USA

**Keywords:** sow, selenium yeast, litter size, embryo implantation, antioxidant capacity, gut microbiota

## Abstract

Early embryonic implantation quality is a key determinant of litter performance in sows. Selenium plays a vital role in maintaining optimal female reproductive performance. This study aims to assess the impact of selenium yeast supplementation during early pregnancy on embryo implantation efficiency and litter size in sows. This trial involved multiparous Landrace × Yorkshire (L × Y) crossbred sows. The control group (*n* = 45) was fed a basal diet. The selenium yeast group (*n* = 43) was supplemented with selenium yeast from 7 days before mating through gestation day 21. The results showed that selenium yeast supplementation significantly increased litter size (*P* < 0.05). Selenium yeast supplementation significantly increased serum progesterone and enhanced antioxidant capacity, as evidenced by elevated serum glutathione peroxidase, superoxide dismutase, and catalase activities, along with reduced malondialdehyde activity (*P* < 0.05). 16S rRNA gene sequencing and untargeted metabolomic analysis revealed that supplementation with selenium yeast significantly increased the abundances of *Lactobacillus amylovorus* (*L. amylovorus*), *Limosilactobacillus reuteri* (*L. reuteri*), and *Lactobacillus johnsonii* (*L. johnsonii*), as well as the butyric acid levels (*P* < 0.05). Correlation analysis indicated that butyric acid levels were significantly and positively correlated with litter size and healthy litter size (*P* < 0.05). In sum, the increase in litter size resulting from selenium yeast supplementation during early pregnancy is associated with improved maternal antioxidant defenses, enhanced progesterone production, modulation of gut microbiota composition, and alterations in metabolic profiles in sows.

**IMPORTANCE:**

This study demonstrated that selenium yeast supplementation during early pregnancy plays a critical role in reproductive performance. It improves embryo implantation quality and increases litter size. Systematic evaluation of selenium yeast supplementation revealed that it significantly enhances maternal antioxidant capacity and progesterone concentrations. Furthermore, it promotes the enrichment of beneficial *Lactobacillus* species and increases butyric acid levels. Further analysis revealed that butyric acid levels were significantly positively correlated with litter size and healthy litter size, suggesting that *Lactobacillus amylovorus*, *Limosilactobacillus reuteri*, *Lactobacillus johnsonii*, and butyric acid may be potential probiotics and metabolites regulating litter size in sows. The study provides scientific evidence that precision nutrition strategies can improve sow reproductive performance and herd productivity. It also holds significant practical value for sow health management and the swine industry.

## INTRODUCTION

Most embryo loss in mammals occurs during the peri-implantation period; the quality of embryo implantation is a key determinant of both embryonic survival and final litter size (1, 2). Among livestock, sows exhibit the highest rates of early embryonic loss, with approximately 30%–50% of embryos lost during early gestation (2). Pregnancy is a period in which the mother continuously experiences oxidative stress; stress responses are triggered by estrus, pregnancy, parturition, and lactation, as well as passive stress from fetal hormone secretion and intrauterine stress due to uterine tension and the intrauterine environment (3). Oxidative stress can damage embryos and the endometrium, potentially causing implantation failure (4). Notably, selenium exerts antioxidant effects that reduce oxidative stress in the uterine microenvironment, thereby mitigating its adverse impact on embryo survival and development (5, 6). Selenium exists in two major forms: inorganic and organic selenium. Inorganic selenium supplementation may pose toxicity risks (7). In contrast, organic selenium exhibits higher bioavailability, owing to improved gastrointestinal absorption and a higher toxicity threshold (8, 9). The dietary selenium requirement for pigs is 0.15–0.4 mg/kg, determined by the saturation of glutathione peroxidase (GSH-Px) activity in blood and tissues and by indicators of health and physiological status (10). Dietary selenium concentrations exceeding 0.4 mg/kg are generally considered to surpass the nutritional requirements of many species and may be wasteful or potentially hazardous to animals and the environment (10).

Gut microbiota is closely associated with pregnancy outcomes and plays a crucial role in maintaining maternal physiology and supporting fetal development (2, 11). Changes in gut microbiota composition and abundance, along with metabolite translocation to the brain, ovaries, uterus, and placenta during pregnancy, may disrupt the maternal reproductive endocrine system and modulate epigenetic modifications in embryonic and fetal development (12–14). Selenium may shape the gut microbiota by modulating its diversity and richness, while increasing the relative abundance of beneficial microbiota (15). Improving sow reproductive performance reduces the demand for land, labor, and feed. Consequently, numerous studies have explored nutritional interventions to enhance reproductive outcomes, particularly healthy litter size. Current research suggests that selenium supplementation positively affects reproductive performance (16). However, it remains unclear whether early pregnancy supplementation can enhance embryo implantation and increase litter size. Therefore, this study systematically evaluates the effects of selenium yeast supplementation, providing a total of 0.40 mg Se/kg diet during early pregnancy, on sow reproductive performance, reproductive hormones, antioxidant capacity, gut microbiota, and metabolic profiles.

## RESULTS

### Impact of selenium yeast supplementation on sow reproductive performance

**Fig 1:**
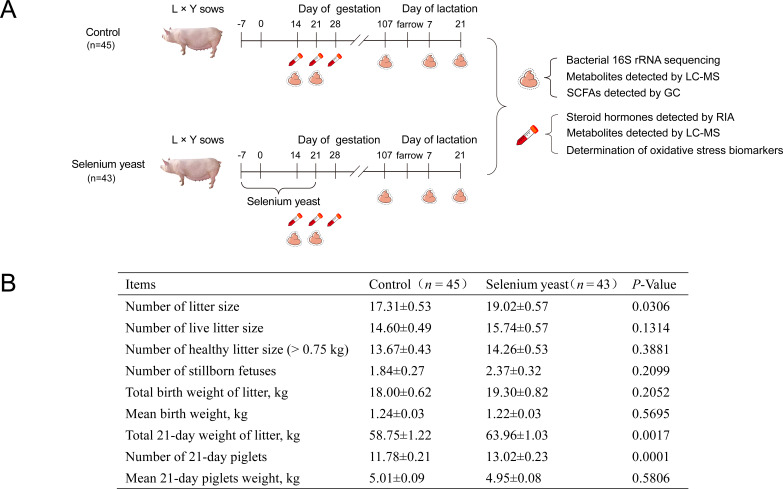
Overview of the experimental design and the comparative analysis of reproductive performance between two groups. (**A**) Overview of the experimental design. The control group included 45 sows, and the selenium yeast group included 43 sows. (**B**) Analysis of differences in reproductive performance between control and selenium yeast groups. Statistical significance between groups was determined by unpaired Student’s *t*-test. All data were presented as mean ± standard error of the mean (SEM). *P* values < 0.05 were considered statistically significant.

The experimental design is illustrated in Fig. 1A. The reproductive performance of the two groups is summarized in Fig. 1B. Litter size was significantly greater in the selenium yeast group than in the control group (*P* < 0.05). The selenium yeast group exhibited significantly higher total litter weight at lactation 21 days (*P* < 0.01) and a greater number of piglets at lactation 21 days (*P* < 0.001) compared with the control group.

### Differences in intestinal microbiota composition between control and selenium yeast-supplemented sows on gestation days 14 and 21

**Fig 2:**
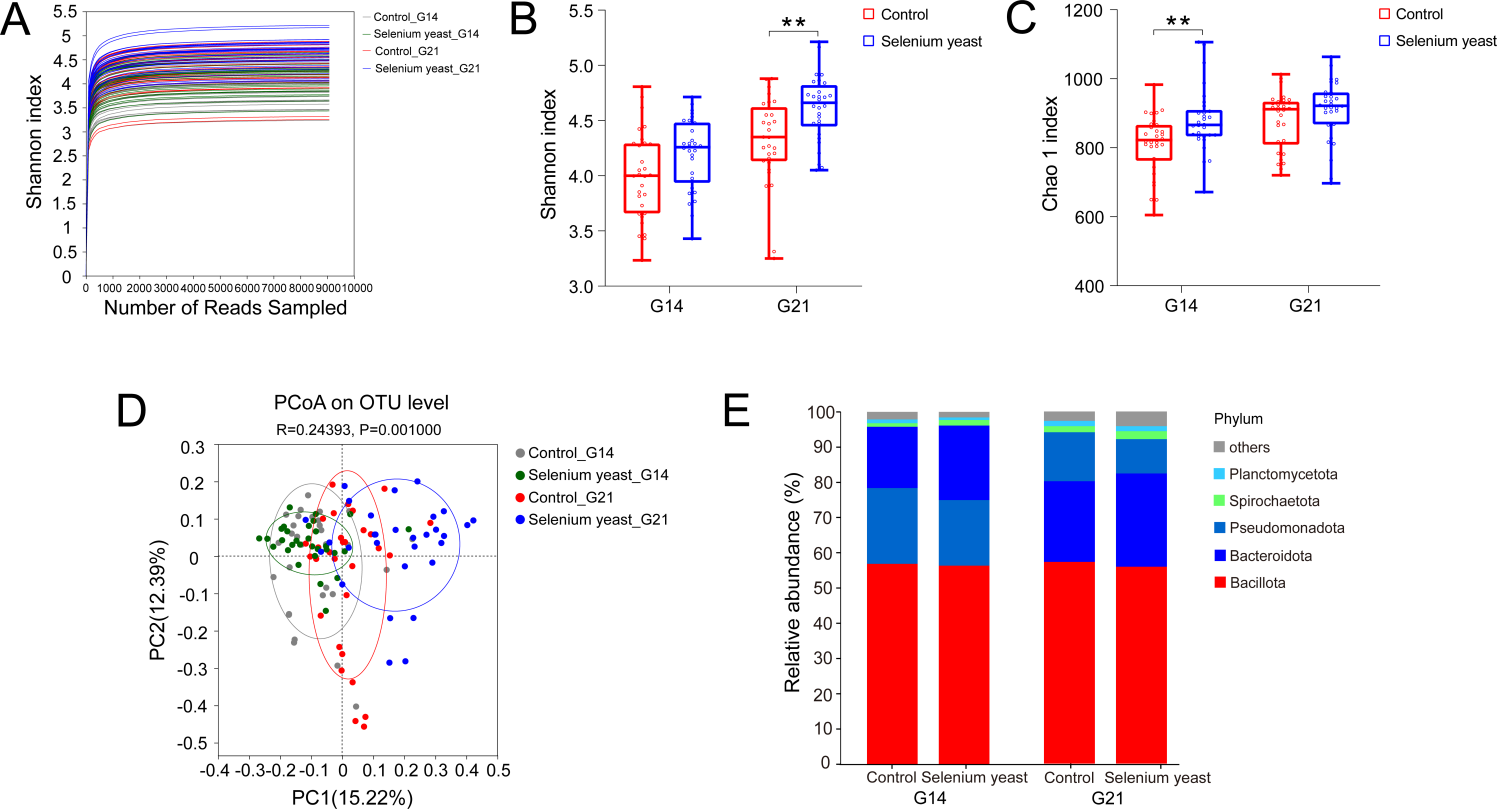
Comparison of intestinal microbiota composition between control and selenium yeast-supplemented sows on gestation days 14 and 21. (**A**) Rarefaction curves of bacteria between control and selenium yeast supplemented sows, with all samples rarefied to 9,075 sequences per sample prior to analysis. Comparison of microbial community diversity, measured by the Shannon index (**B**) and Chao1 index (**C**), between control and selenium yeast-supplemented sows. (**D**) PCoA based on Bray–Curtis distances of operational taxonomic unit (OTU) profiles between control and selenium yeast-supplemented sows. (**E**) Fecal microbial community composition at the phylum level in control and selenium yeast-supplemented sows. Statistical analyses were performed using the Mann–Whitney U test (**B and C**). **P < 0.01. *n* = 30.

**Fig 3:**
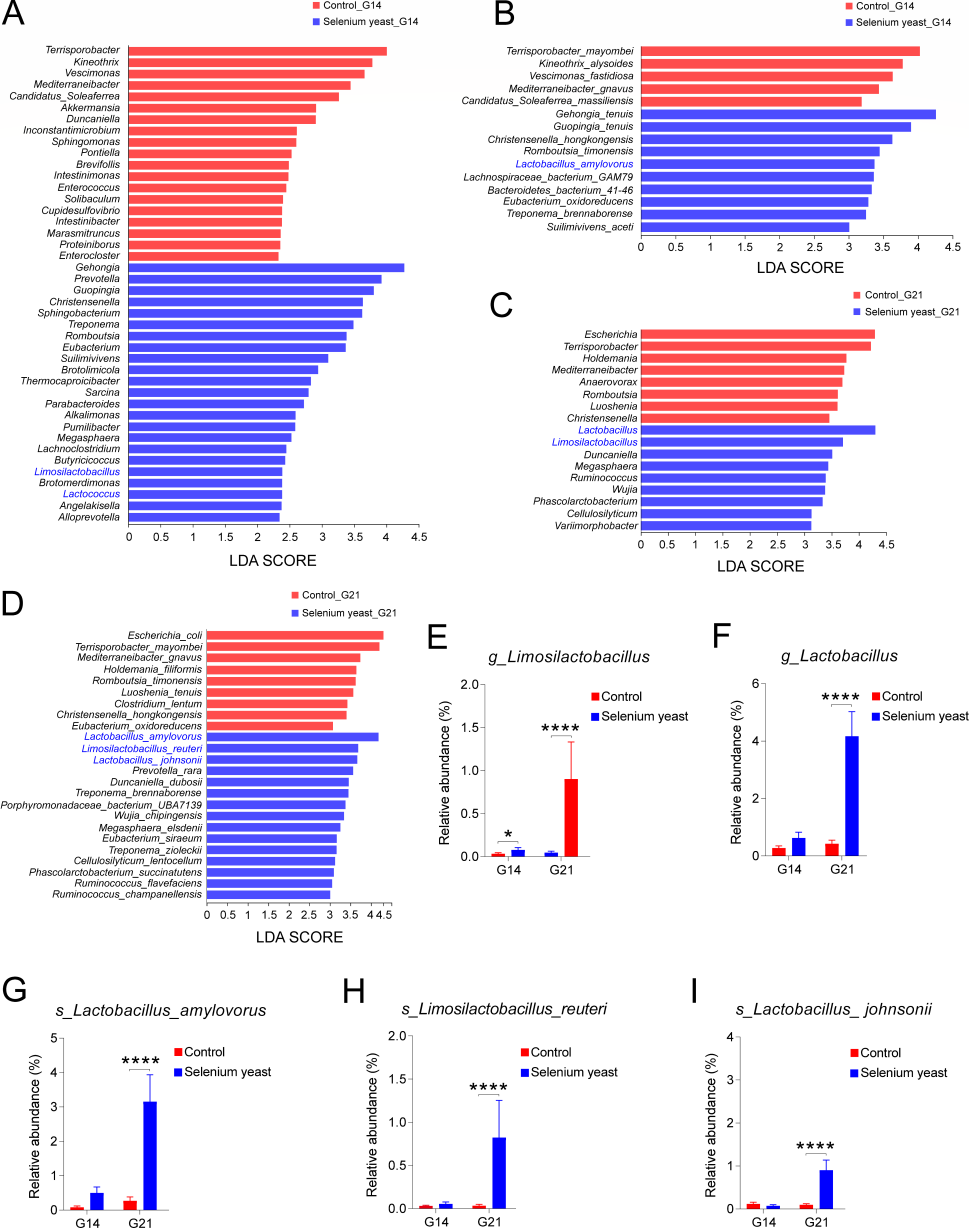
Comparison of intestinal microbial community differences between control and selenium yeast-supplemented sows on gestation days 14 and 21. Differential abundance analysis at the genus (**A**) and species (**B**) levels between control and selenium yeast-supplemented sows on gestation day 14. Analysis of differential microbiota at the genus (**C**) and species (**D**) levels between control and selenium yeast-supplemented sows on gestation day 21. (**E–I**) Comparison of the relative abundances of target genera (*Limosilactobacillus* and *Lactobacillus*) and species (*L. amylovorus*, *L. reuteri*, and *L. johnsonii*) between control and selenium yeast-supplemented sows. Statistical analyses were performed using the Mann–Whitney U test (**E–I**). *P < 0.05, ****P < 0.0001. *n* = 30.

To identify functional microbiota that may influence embryo survival, we performed 16S rRNA gene sequencing on fecal samples collected from sows during early gestation. Rarefaction curves indicated that the sequencing depth was sufficient to capture nearly all bacterial taxa (Fig. 2A). Alpha diversity analysis showed that the Shannon index was significantly higher in selenium yeast supplemented sows on gestation day 21, indicating increased microbial diversity (*P* < 0.01) (Fig. 2B). The Chao1 index was significantly higher in selenium yeast supplemented sows on gestation day 14, suggesting greater community abundance (*P* < 0.01) (Fig. 2C). Principal coordinates analysis (PCoA) based on Bray–Curtis distances revealed a clear separation between the two groups on gestation days 14 and 21 (*P* = 0.01) (Fig. 2D). At the phylum level, *Bacillota*, *Bacteroidota*, and *Pseudomonadota* were the dominant phyla on gestation days 14 and 21 (Fig. 2E). At the genus-species levels, linear discriminant analysis effect size (LEfSe) revealed significantly higher relative abundances of *Limosilactobacillus*, *Lactobacillus*, and *Lactobacillus amylovorus* (*L. amylovorus*) in selenium yeast supplemented sows on gestation day 14 (*P* < 0.05) (Fig. 3A and B). On gestation day 21, LEfSe analysis showed significantly higher relative abundances of *Limosilactobacillus*, *Lactobacillus*, *L. amylovorus*, *Limosilactobacillus reuteri* (*L. reuteri*), and *Lactobacillus johnsonii* (*L. johnsonii*) in the selenium yeast supplemented sows (*P* < 0.05) (Fig. 3C and D). Further analysis showed that *Limosilactobacillus* relative abundance was significantly higher in the selenium yeast supplemented sows on gestation days 14 (*P* < 0.05) and 21 (*P* < 0.0001) (Fig. 3E). Similarly, *Lactobacillus* relative abundance was significantly higher in the selenium yeast supplemented sows on gestation day 21 (*P* < 0.0001) (Fig. 3F). *L. amylovorus* (Fig. 3G), *L. reuteri* (Fig. 3H), and *L. johnsonii* (Fig. 3I) were also significantly more abundant in selenium yeast supplemented sows on gestation day 21 (*P* < 0.0001).

### Alterations in the metabolic profiles of the intestine and blood in selenium yeast-supplemented sows compared with controls on gestation days 14 and 21

**Fig 4:**
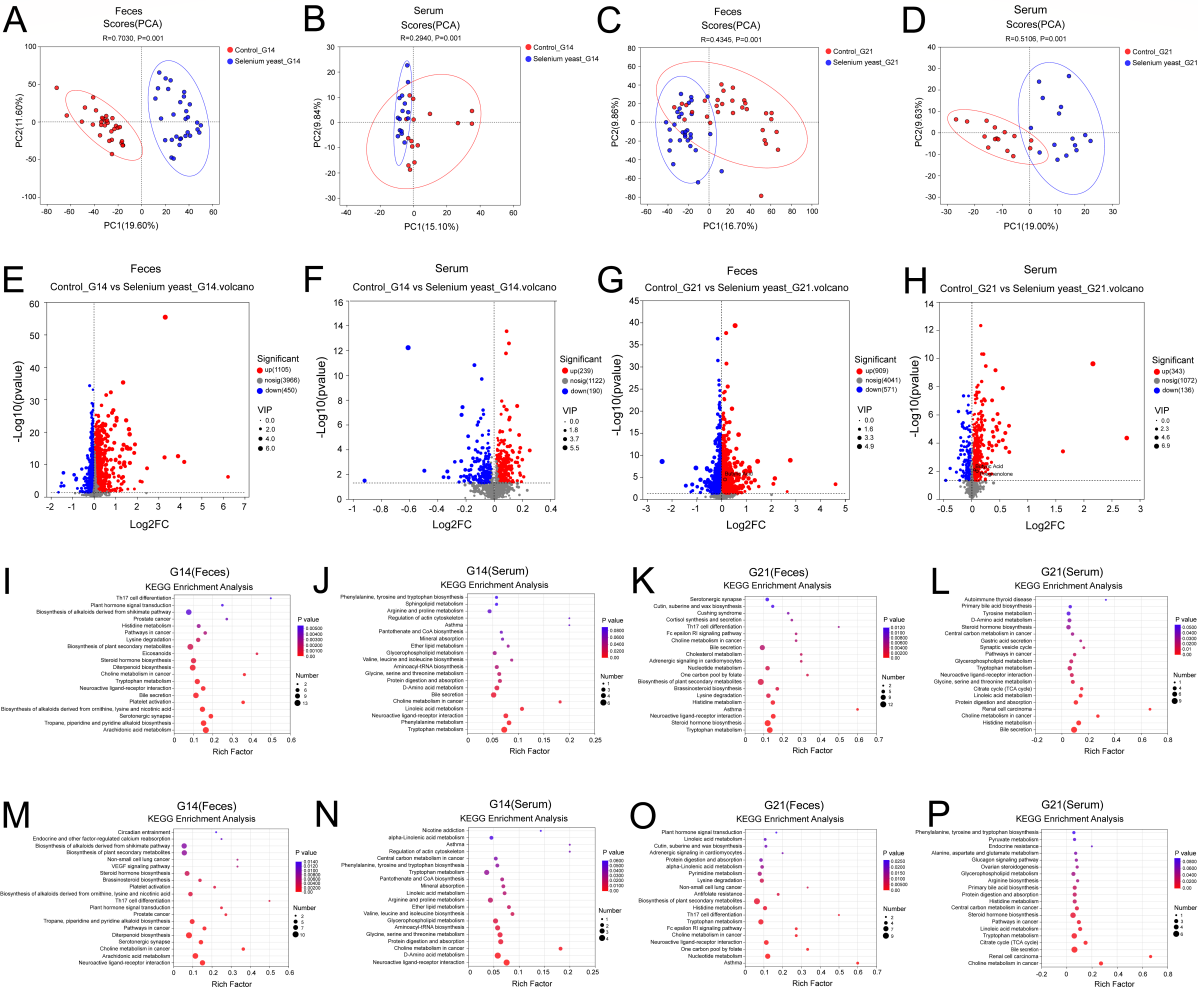
Alterations in intestinal and blood metabolic profiles between control and selenium yeast-supplemented sows on gestation days 14 and 21. PCA of fecal (**A**) and serum (**B**) metabolomes between control and selenium yeast-supplemented sows on gestation day 14. PCA of fecal (**C**) and serum (**D**) metabolomes between control and selenium yeast supplemented sows on gestation day 21. Volcano plot analysis of fecal (**E**) and serum (**F**) metabolomes between control and selenium yeast-supplemented sows on gestation day 14. Volcano plot analysis of fecal (**G**) and serum (**H**) metabolomes between control and selenium yeast supplemented sows on gestation day 21. KEGG pathway enrichment analysis of fecal (**I**) and serum (**J**) differential metabolites between control and selenium yeast supplemented sows on gestation day 14. KEGG pathway enrichment analysis of fecal (**K**) and serum (**L**) differential metabolites between control and selenium yeast-supplemented sows on gestation day 21. KEGG pathway enrichment analysis of fecal (**M**) and serum (**N**) differentially upregulated metabolites between control and selenium yeast-supplemented sows on gestation day 14. KEGG pathway enrichment analysis of fecal (**O**) and serum (**P**) differentially upregulated metabolites between control and selenium yeast-supplemented sows on gestation day 21. Color gradients and circle sizes, respectively, correspond to the fluctuation of the *P* value and impacts. *n* = 30 (fecal metabolomes), *n* = 15 (serum metabolomes).

Metabolic regulation plays a critical role in microbiota-mediated reproductive processes in mammals. Therefore, fecal and serum untargeted metabolomics sequencing was conducted. Principal component analysis (PCA) of fecal and serum samples collected on gestation days 14 and 21 demonstrated clear separation between control and selenium yeast supplemented sows (*P* = 0.01) (Fig. 4A through D). Orthogonal partial least squares discriminant analysis (OPLS-DA) was used to identify differential metabolites between the two groups. The OPLS-DA model was validated with 200 permutation tests to ensure the absence of overfitting (Fig. S1A through H). On gestation day 14, fecal samples from the selenium yeast group showed 1,105 significantly upregulated and 450 significantly downregulated metabolites compared with controls (*P* < 0.05) (Fig. 4E). Similarly, serum samples on gestation day 14 showed 239 significantly upregulated and 190 significantly downregulated metabolites in the selenium yeast group compared with controls (*P* < 0.05) (Fig. 4F). On gestation day 21, fecal samples revealed 909 significantly upregulated and 571 significantly downregulated metabolites in the selenium yeast group versus controls (*P* < 0.05) (Fig. 4G). Serum samples on gestation day 21 showed 343 significantly upregulated and 136 significantly downregulated metabolites in the selenium yeast group compared with controls (*P* < 0.05) (Fig. 4H). KEGG enrichment analysis of differential metabolites from fecal and serum samples collected on gestation days 14 and 21 revealed significant enrichment in steroid hormone biosynthesis pathways between control and selenium yeast supplemented sows (*P* < 0.05) (Fig. 4I, K, and L). Untargeted metabolites were also significantly enriched in steroid hormone biosynthesis pathways between control and selenium yeast supplemented sows (*P* < 0.05) (Fig. 4M and P).

### Impact of selenium yeast supplementation on selenium levels, reproductive hormones, and antioxidant-related indicators in sows

**Fig 5:**
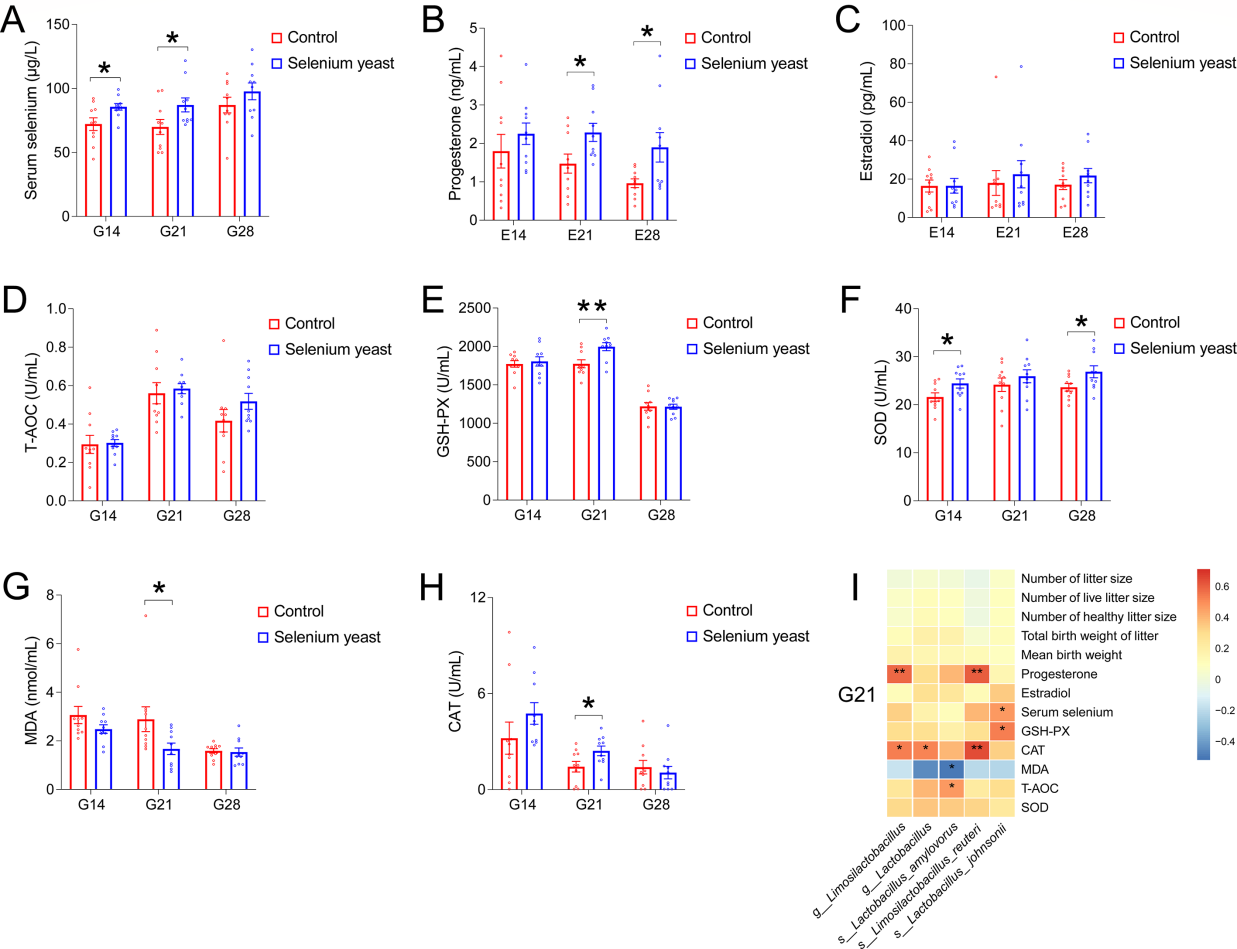
Impact of selenium yeast supplementation on selenium levels, reproductive hormones, and antioxidant capacity in sows. (**A**) Levels of selenium in serum. Levels of progesterone (**B**) and estradiol (**C**) in serum. Levels of T-AOC (**D**), GSH-Px (**E**), SOD (**F**), MDA (**G**), and CAT (**H**) in serum. (**I**) Correlation among intestinal microbiota, reproductive performance, selenium levels, reproductive hormones, and antioxidant-related indicators on gestation day 21. Correlation coefficients were analyzed using Spearman correlation analysis. Red color indicates positive correlation, and blue color indicates negative correlation. Statistical significance between groups was determined by unpaired Student’s *t*-tests (**A–H**). All data were presented as mean ± SEM (**A–H**). *P < 0.05, **P < 0.01. *n* = 10.

To evaluate the sustained effects of selenium yeast supplementation on early pregnancy, we measured serum selenium levels, estradiol and progesterone concentrations, as well as antioxidant-related indicators on gestation days 14, 21, and 28. Serum selenium levels were significantly higher in the selenium yeast group than in the control group on gestation days 14 and 21 (*P* < 0.05) (Fig. 5A). Serum progesterone concentrations were significantly higher in the selenium yeast group on gestation days 21 and 28 (*P* < 0.05), while estradiol levels remained unchanged (Fig. 5B and C). Serum total antioxidant capacity (T-AOC) activities tended to be higher in the selenium yeast group than in the control group on gestation days 21 and 28 (Fig. 5D). Serum GSH-Px activities were significantly higher in the selenium yeast group than in the control group on gestation day 21 (*P* < 0.01) (Fig. 5E). Serum superoxide dismutase (SOD) activities were significantly higher in the selenium yeast group than in the control group on gestation days 14 and 28 (*P* < 0.05) (Fig. 5F). Serum malondialdehyde (MDA) activities were significantly lower in the selenium yeast group than in the control group on gestation day 21 (*P* < 0.05) (Fig. 5G). Serum catalase (CAT) activities were significantly higher in the selenium yeast group than in the control group on gestation day 21 (*P* < 0.05) (Fig. 5H).

### Spearman correlation analysis was performed to assess relationships among intestinal microbiota, metabolites, reproductive performance, selenium levels, reproductive hormones, and antioxidant-related indicators

**Fig 6:**
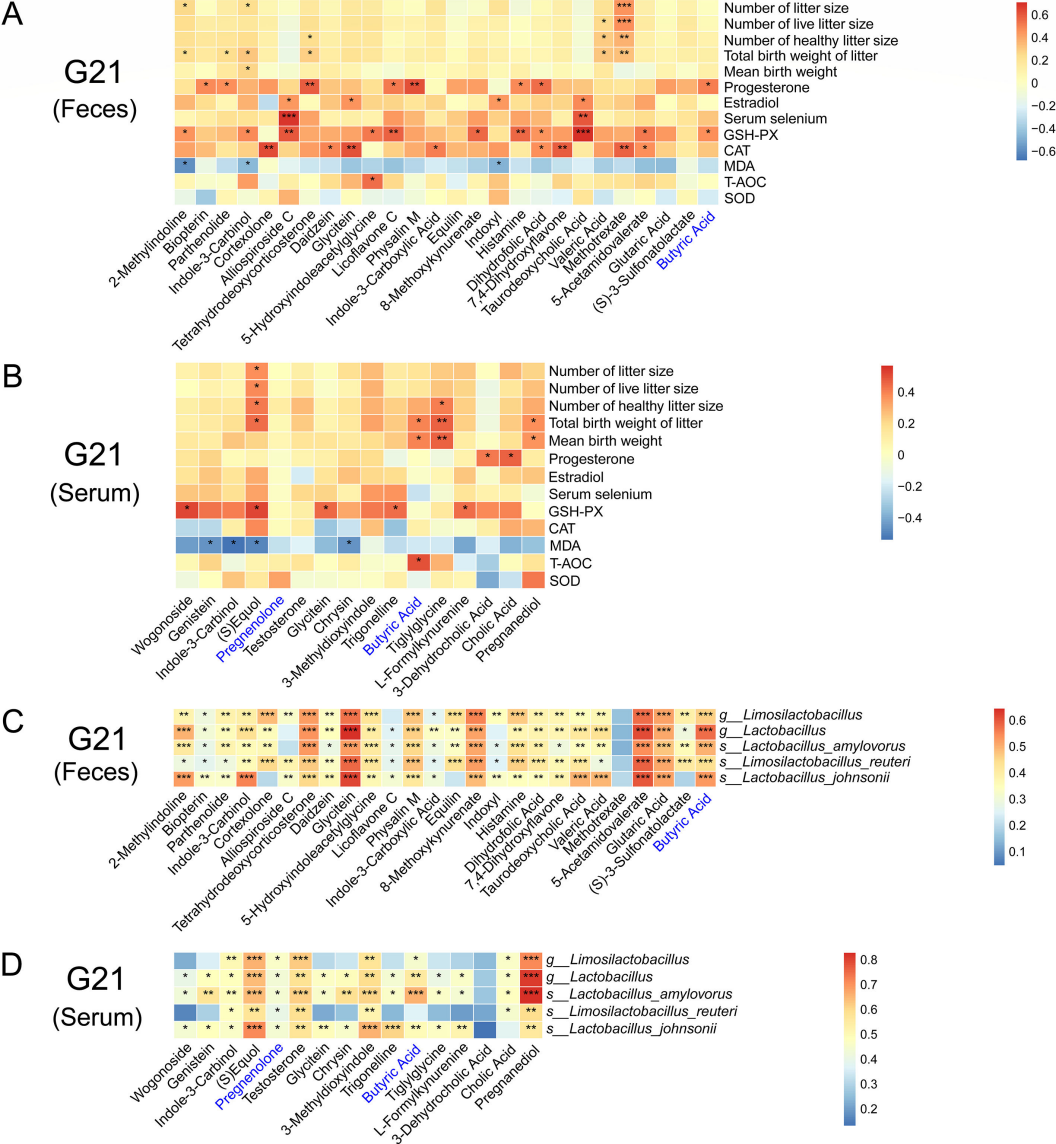
Correlations among metabolites, reproductive performance, selenium levels, reproductive hormones, antioxidant-related indicators, and intestinal microbiota. (**A**) Correlation among metabolites of fecal sample, reproductive performance, selenium levels, reproductive hormones, and antioxidant-related indicators on gestation day 21. (**B**) Correlation among metabolites of serum sample, reproductive performance, selenium levels, reproductive hormones, and antioxidant-related indicators on gestation day 21. (**C**) Correlation between intestinal microbiota and metabolites of fecal sample on gestation day 21. (**D**) Correlation between intestinal microbiota and metabolites of the serum sample on gestation day 21. Correlation coefficients were analyzed using Spearman correlation analysis. Red color indicates positive correlation, and blue color indicates negative correlation.

*Limosilactobacillus* and *L. reuteri* abundances were significantly and positively correlated with serum progesterone concentrations and CAT activity (*P* < 0.05). Similarly, *Lactobacillus* abundance was significantly and positively correlated with serum CAT activity (*P* < 0.05). *L. johnsonii* abundance was significantly and positively associated with serum selenium and GSH-Px activity (*P* < 0.05). *L. amylovorus* abundance was significantly and positively correlated with serum T-AOC activity and negatively correlated with MDA activity (*P* < 0.05) (Fig. 5I). On gestation day 21, butyric acid levels were significantly and positively correlated with serum progesterone concentrations and GSH-Px activity (*P* < 0.05) (Fig. 6A). On gestation day 21, serum butyric acid levels showed significant positive correlations with total and mean litter birth weights and T-AOC activity (*P* < 0.05) (Fig. 6B). On gestation day 21, the relative abundances of *Limosilactobacillus*, *Lactobacillus*, *L. amylovorus*, *L. reuteri*, and *L. johnsonii* were significantly and positively correlated with multiple metabolites, including 2-methylindoline, indole-3-carbinol, tetrahydrodeoxycorticosterone, glycitein, physalin M, 8-methoxykynurenate, 5-acetamidovalerate, glutaric acid, and butyric acid (*P* < 0.05) (Fig. 6C). In serum samples on gestation day 21, the same bacterial taxa showed significant positive correlations with indole-3-carbinol, (S)-equol, pregnenolone, testosterone, 3-methyldioxyindole, butyric acid, and pregnanediol (*P* < 0.05). Among these metabolites, pregnenolone, testosterone, and pregnanediol are enriched in the steroid hormone biosynthesis pathway (Fig. 6D).

### Differences in intestinal microbiota composition between control and selenium yeast-supplemented sows on gestation day 107 and lactation days 7 and 21

**Fig 7:**
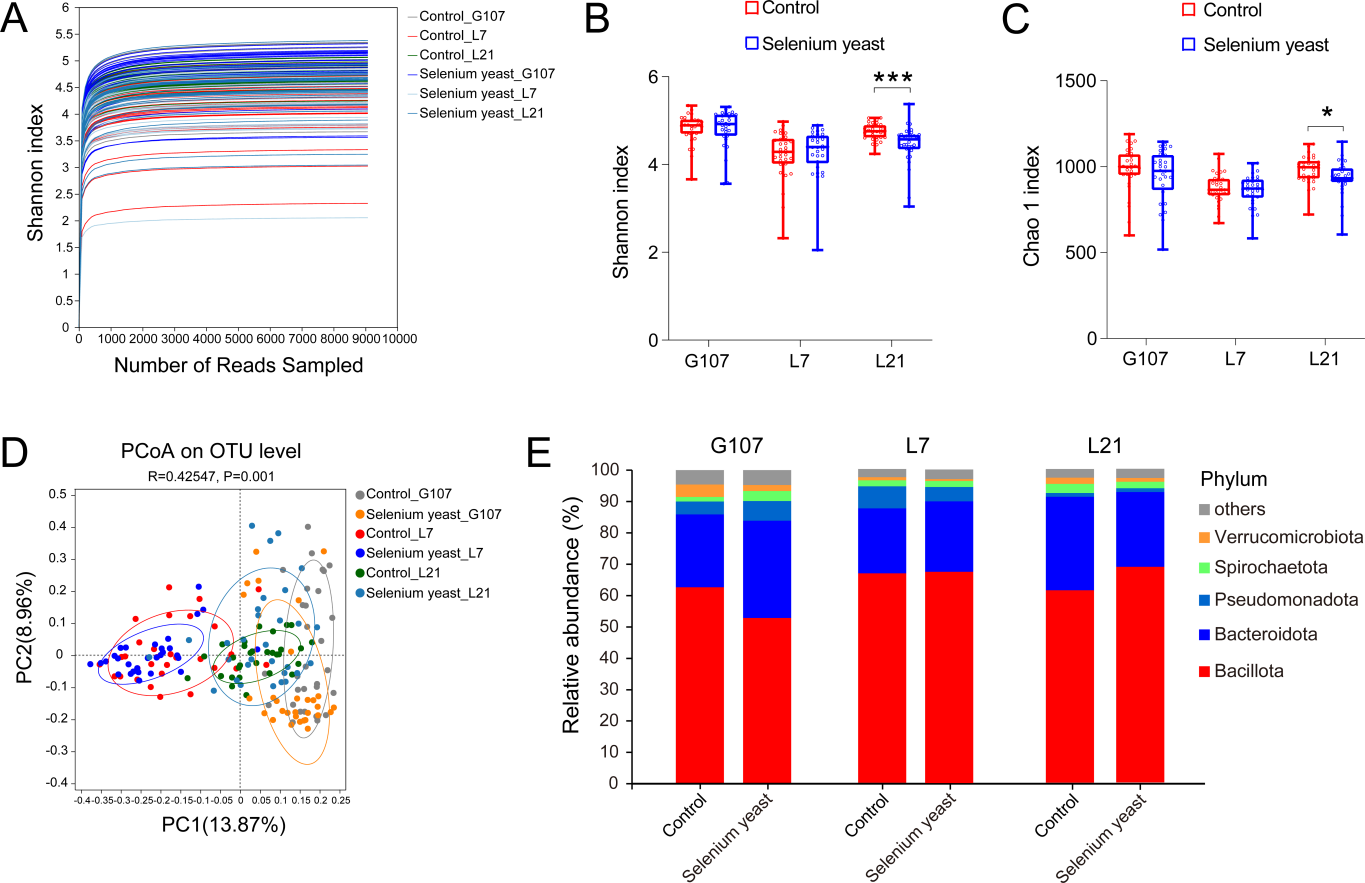
Comparison of intestinal microbiota composition between control and selenium yeast-supplemented sows on gestation day 107 and lactation days 7 and 21. (**A**) Rarefaction curves of bacteria between control and selenium yeast-supplemented sows, with all samples rarefied to 9,075 sequences per sample prior to analysis. Comparison of microbial community diversity, measured using the Shannon index (**B**) and Chao1 index (**C**), between control and selenium yeast-supplemented sows. (**D**) PCoA based on Bray–Curtis distances of OTU profiles between control and selenium yeast-supplemented sows. (**E**) Fecal microbial community composition at the phylum level in control and selenium yeast-supplemented sows. Statistical analyses were performed using the Mann–Whitney *U* test (**B and C**). *P < 0.05, ***P < 0.001. *n* = 30.

To evaluate the sustained effects of selenium yeast supplementation on the gut microbiota, 16S rRNA gene sequencing was performed to compare the intestinal microbiota composition between control and selenium yeast-supplemented sows on gestation day 107 and lactation days 7 and 21. Rarefaction curves indicated that the sequencing depth was sufficient to capture nearly all bacterial taxa (Fig. 7A). Alpha diversity analysis revealed differences in microbial diversity, as measured by the Shannon index, between the two groups (Fig. 7B). Differences in microbial richness, as estimated by the Chao1 index, were also observed between the two groups (Fig. 7C). PCoA based on Bray–Curtis distances revealed a clear separation between the two groups across different time points (*P* = 0.01) (Fig. 7D). At the phylum level, *Bacillota* and *Bacteroidota* were the dominant phyla at all time points (Fig. 7E). At the genus-species levels, LEfSe analysis revealed a significantly higher relative abundance of *L. reuteri* in selenium yeast-supplemented sows specifically on lactation day 7 (*P* < 0.05) (Fig. S2D). These findings suggest that the stable colonization of *Limosilactobacillus*, *Lactobacillus*, *L. amylovorus*, *L. reuteri*, and *L. johnsonii* may depend on selenium yeast supplementation (Fig. S2A through F).

### Alterations in the metabolic profiles of intestine in selenium yeast-supplemented sows compared with controls on gestation day 107 and lactation days 7 and 21

**Fig 8:**
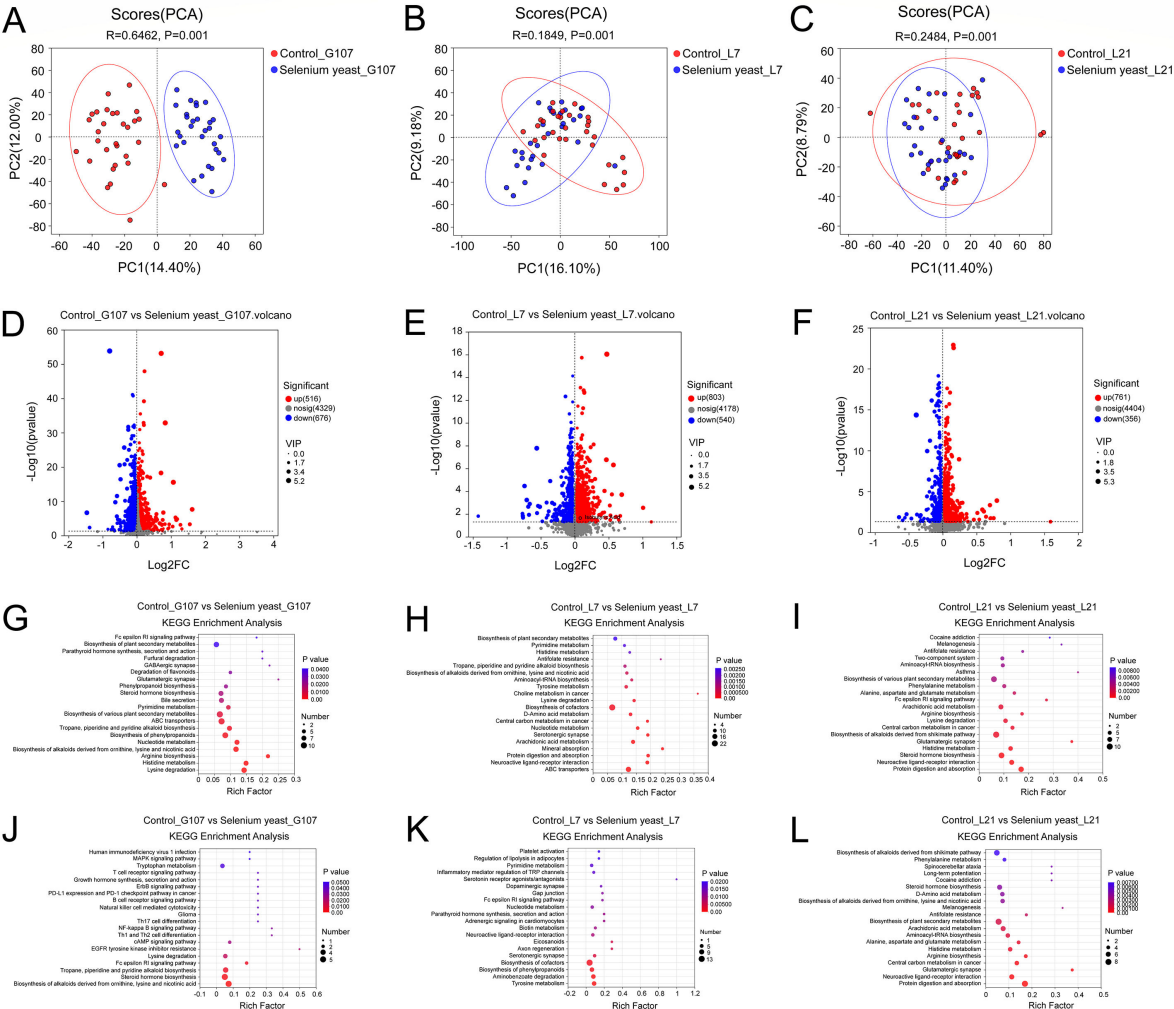
Alterations in intestinal metabolic profiles between control and selenium yeast-supplemented sows on gestation day 107 and lactation days 7 and 21. PCA of fecal metabolomes between control and selenium yeast-supplemented sows on gestation day 107 (**A**) and lactation days 7 (**B**) and 21 (**C**). Volcano plot analysis of fecal metabolomes between control and selenium yeast-supplemented sows on gestation day 107 (**D**) and lactation days 7 (**E**) and 21 (**F**). KEGG pathway enrichment analysis of fecal differential metabolites between control and selenium yeast-supplemented sows on gestation day 107 (**G**) and lactation days 7 (**H**) and 21 (**I**). KEGG pathway enrichment analysis of fecal differentially upregulated metabolites between control and selenium yeast-supplemented sows on gestation day 107 (**J**) and lactation days 7 (**K**) and 21 (**L**). Color gradients and circle sizes, respectively, correspond to the fluctuation of the *P* value and impacts. *n* = 30 (fecal metabolomes).

PCA of fecal samples collected on gestation day 107 and lactation days 7 and 21 showed clear separation between control and selenium yeast groups (*P* = 0.01) (Fig. 8A through C). OPLS-DA was used to identify differential metabolites between the two groups. The OPLS-DA model was validated with 200 permutation tests to ensure the absence of overfitting (Fig. S1I through N). On gestation day 107, 516 metabolites were significantly upregulated and 676 metabolites were significantly downregulated in the selenium yeast group compared with the control group (*P* < 0.05) (Fig. 8D). On lactation day 7, 803 metabolites were significantly upregulated and 540 metabolites were significantly downregulated in the selenium yeast group compared with the control group (*P* < 0.05) (Fig. 8E). On lactation day 21, 761 metabolites were significantly upregulated and 356 metabolites were significantly downregulated in the selenium yeast group compared with the control group (*P* < 0.05) (Fig. 8F). KEGG enrichment analysis of differential metabolites from fecal samples collected on gestation day 107 and lactation day 21 revealed significant enrichment in steroid hormone biosynthesis between control and selenium yeast-supplemented sows (*P* < 0.05) (Fig. 8G and I). Upregulated metabolites were also significantly enriched in steroid hormone biosynthesis between control and selenium yeast-supplemented sows (*P* < 0.05) (Fig. 8J and L).

### Determination of SCFAs using gas chromatography and analysis of their correlations with intestinal microbiota and phenotypic traits

**Fig 9:**
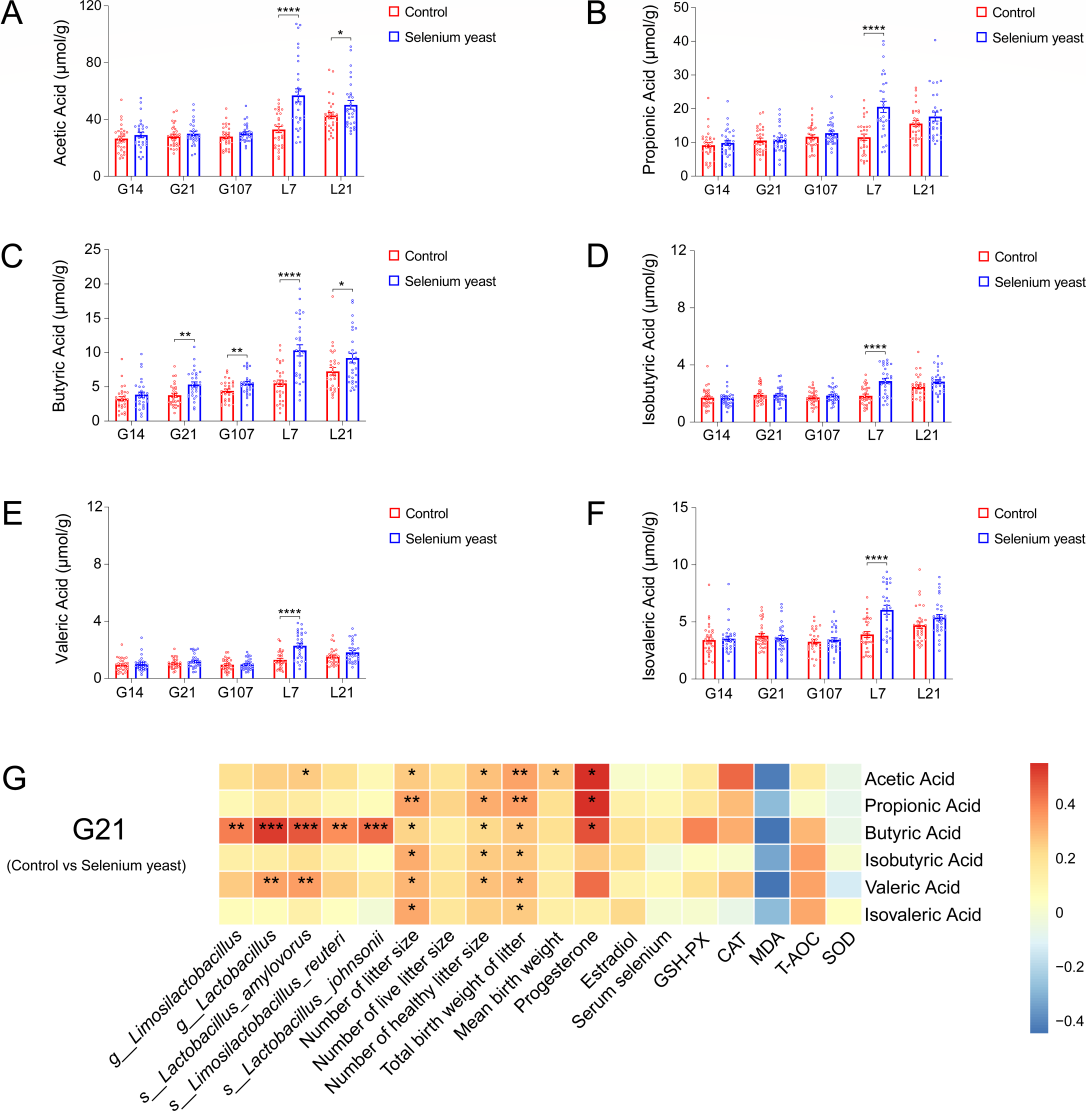
Levels of SCFAs in sow feces and their correlations with intestinal microbiota and phenotypic traits. Levels of acetic acid (**A**), propionic acid (**B**), butyric acid (**C**), isobutyric acid (**D**), valeric acid (**E**), and isovaleric acid (**F**) in fecal samples from control and selenium yeast-supplemented sows on gestation days 14, 21, and 107, and lactation days 7 and 21 (*n* = 30). (**G**) Correlations among SCFAs, intestinal microbiota, and phenotypic traits on gestation day 21. Correlation coefficients were analyzed using Spearman correlation analysis. Red color indicates positive correlation, and blue color indicates negative correlation. Statistical significance between groups was determined by unpaired Student’s *t*-tests. All data were presented as mean ± SEM. *P < 0.05, **P < 0.01, ****P < 0.0001.

To quantify short-chain fatty acids (SCFAs) levels in sow feces at multiple time points during gestation and lactation, we used gas chromatography for SCFAs measurement. On lactation day 7, the selenium yeast group showed significantly higher levels of acetic acid and propionic acid than the control group (*P* < 0.0001) (Fig. 9A and B). On lactation day 21, acetic acid levels were significantly higher in the selenium yeast group than in the control group (*P* < 0.05) (Fig. 9A). Butyric acid levels were significantly higher in the selenium yeast group than in the control group on gestation days 21 (*P* < 0.01) and 107 (*P* < 0.01) and on lactation days 7 (*P* < 0.0001) and 21 (*P* < 0.05) (Fig. 9C). On lactation day 7, isobutyric acid levels were significantly higher in the selenium yeast group than in the control group (*P* < 0.0001) (Fig. 9D). On lactation day 7, valeric acid levels were significantly higher in the selenium yeast group than in the control group (*P* < 0.0001) (Fig. 9E). On lactation day 7, isovaleric acid levels were significantly higher in the selenium yeast group than in the control group (*P* < 0.0001) (Fig. 9F). Spearman correlation analysis was performed to assess the relationships among SCFAs, intestinal microbiota, and phenotypic traits. On gestation day 21, the relative abundances of *Limosilactobacillus*, *Lactobacillus*, *L. amylovorus*, *L. reuteri*, and *L. johnsonii* were significantly and positively correlated with butyric acid levels (*P* < 0.05) (Fig. 9G). Butyric acid levels were significantly and positively correlated with progesterone concentrations, litter size, healthy litter size, and total birth weight litter (*P* < 0.05) (Fig. 9G).

## DISCUSSION

Our findings indicate that selenium yeast supplementation during early pregnancy significantly increases litter size. Progesterone plays a central role in coordinating interactions among the embryo, ovaries, and uterus during early pregnancy by inducing secretory changes in the endometrium, which are essential for successful implantation and enhance endometrial receptivity to the blastocyst (17, 18). The results showed that serum progesterone concentrations on gestation day 21 were significantly higher in the selenium yeast group than in the control group. Cerny et al. reported that selenium supplementation in dairy cows enhances progesterone production, which aligns with our findings (19). Untargeted serum metabolomic analysis revealed that pregnenolone levels were significantly elevated in the selenium yeast group on gestation day 21. As a direct precursor of progesterone, the elevated pregnenolone concentrations may enhance progesterone synthesis (20). These results further support the observed increase in serum progesterone concentrations. In addition, accumulating evidence indicates that the balance between antioxidants and prooxidants is critical for regulating mammalian reproductive processes, including follicular development, ovulation, fertilization, corpus luteum formation, embryo implantation, and early placental growth (21). Accordingly, we examined serum antioxidant-related indicators during early pregnancy. Selenium yeast supplementation significantly increased serum activities of key antioxidant enzymes (GSH-Px, SOD, and CAT) and decreased the activity of the oxidative damage marker MDA. Selenium is widely recognized for its antioxidant properties, which have been extensively documented in the literature, further supporting our findings (22–24). Collectively, these results suggest that selenium yeast supplementation enhances serum progesterone concentrations and strengthens the maternal antioxidant defense system by stimulating enzymatic antioxidant activity, thereby contributing to improved reproductive outcomes during early gestation.

Moreover, 16S rRNA gene sequencing revealed that selenium yeast supplementation during early pregnancy significantly increased the relative abundance of *L. amylovorus*, *L. reuteri*, and *L. johnsonii* on gestation day 21. Dowley et al. reported that dietary supplementation with mushroom powder rich in organic selenium increased the relative abundance of *Lactobacillus* in the cecum of weaned piglets (25). However, because selenium-enriched mushroom powder contains various bioactive constituents in addition to selenium, including polysaccharides and other phytochemicals, the observed effects cannot be attributed exclusively to selenium. Nevertheless, the consistent increase in *Lactobacillus* abundance observed across both studies suggests that selenium-containing dietary interventions may be associated with the enrichment of beneficial *Lactobacillus* populations in the gut. On gestation day 21, Spearman correlation analysis revealed that *L. amylovorus* abundance was significantly and positively correlated with serum T-AOC and negatively correlated with MDA activity. *L. reuteri* abundance was significantly and positively correlated with CAT activity. *L. johnsonii* abundance was significantly and positively associated with serum selenium and GSH-Px activity. These results indicate that *L. amylovorus*, *L. reuteri*, and *L. johnsonii* are positively associated with antioxidant capacity. Numerous studies have demonstrated that *L. reuteri* and *L. johnsonii* enhance antioxidant capacity and modulate immune function (26, 27). Collectively, our findings suggest that selenium yeast supplementation promotes the colonization of *L. amylovorus*, *L. reuteri*, and *L. johnsonii*, which may enhance maternal antioxidant and immune defenses, thereby contributing to the regulation of embryonic survival during early pregnancy.

Furthermore, untargeted metabolomic analyses of fecal and serum samples showed significantly elevated butyric acid levels on gestation day 21. Gas chromatography-based quantification of fecal SCFAs further confirmed elevated butyric acid levels. Integrated correlation analysis of SCFAs, bacterial abundance, and phenotypic traits showed that *L. amylovorus*, *L. reuteri*, and *L. johnsonii* were positively associated with butyric acid levels. Butyric acid, a major microbial fermentation product, is a key regulator of systemic energy homeostasis and reproductive endocrinology (28, 29). Several studies have shown that *L. reuteri* and *L. johnsonii* are associated with increased butyric acid levels. For example, Duan et al. demonstrated that a high-fat diet significantly reduced fecal and serum butyric acid levels in mice, whereas reintroduction of *L. reuteri* restored butyric acid levels (30). However, the underlying mechanism remains unclear, as the observed increase in butyric acid may result either from direct metabolic activity of *L. reuteri* or from cross-feeding interactions with other butyric acid-producing members of the gut microbiota. Liu et al. demonstrated that colonization of mice with *L. reuteri* significantly restored cecal butyric acid levels and further showed, through GC–MS analysis of culture supernatants after 24 h of incubation, that *L. reuteri* cultures contained significantly higher butyric acid concentrations than the control medium, suggesting that this bacterium can directly produce butyric acid (31). Similarly, Han et al. reported that supplementation with *L. johnsonii* restored fecal butyric acid levels in hyperuricemic mice (32). Consistent with these findings, Hua et al. further demonstrated that *L. johnsonii* promotes intestinal butyric acid production (33). Unlike *L. reuteri*, the effect of *L. johnsonii* on butyric acid accumulation may be mediated indirectly through microbial cross-feeding interactions. Specifically, *L. johnsonii* produces acetic acid, a key metabolic intermediate that can be converted into butyric acid by other gut microorganisms through cross-feeding and cooperative metabolic processes (34). On gestation day 21, Spearman correlation analysis revealed that butyric acid levels were significantly and positively correlated with litter size and healthy litter size. Ye et al. demonstrated that butyric acid enhances endometrial epithelial cell receptivity by increasing histone H3K9 acetylation and activating the H3K9ac/FoxO1/PCNA signaling pathway, thereby promoting cellular processes that support embryo implantation (35). Collectively, these findings indicate that the colonization of *L. reuteri* and *L. johnsonii* may enhance butyric acid production, thereby supporting embryo implantation and maternal reproductive function during early pregnancy. Collectively, these findings suggest that *L. reuteri* and *L. johnsonii* are positively associated with butyric acid production and accumulation. Given the reported roles of butyric acid in endometrial receptivity and reproductive physiology, these bacterial taxa may be involved in microbial-metabolic networks related to reproductive performance during early pregnancy.

Despite the comprehensive multi-omics analyses performed in this study, several limitations should be acknowledged. First, the serum metabolomics analysis was based on a relatively small sample size (*n* = 15), which may have reduced statistical power, increased the likelihood of false-negative results, and limited the detection of subtle metabolic changes. Therefore, these metabolomic findings should be interpreted with caution and validated in larger cohorts to confirm their robustness and reproducibility. Second, KEGG pathway enrichment analysis was conducted using nominal *P* values without applying Benjamini–Hochberg correction for multiple comparisons. Consequently, the identified pathways should be considered exploratory, as some may represent false-positive enrichments. Future studies incorporating larger sample sizes and independent validation data sets are required to verify the biological relevance of these pathways. Third, although significant associations were observed among *Lactobacillus* species, butyric acid concentrations, antioxidant-related indicators, and reproductive traits, the correlation-based analyses used in this study cannot establish causality. Therefore, it remains unclear whether the observed microbial and metabolic changes directly contribute to improved reproductive performance or merely reflect concurrent biological responses to selenium yeast supplementation. Future studies incorporating targeted microbial interventions, metabolite supplementation experiments, and mechanistic approaches are needed to elucidate the causal roles of specific bacterial taxa and metabolites in regulating reproductive outcomes during early pregnancy.

### Conclusions

In sum, our study demonstrates that selenium yeast supplementation during early pregnancy strengthens maternal antioxidant defenses, enhances progesterone production, modulates gut microbiota composition, and alters metabolic profiles. Collectively, these effects establish a uterine microenvironment conducive to embryo implantation, potentially enhancing embryo attachment and increasing litter size. Notably, selenium yeast supplementation during early pregnancy significantly increased the relative abundance of *L. amylovorus*, *L. reuteri*, and *L. johnsonii*, along with fecal and serum butyric acid levels. *L. amylovorus*, *L. reuteri*, *L. johnsonii*, and butyric acid may serve as critical probiotics and bioactive metabolites that promote litter size, highlighting their potential to improve maternal and embryonic outcomes during early gestation.

## MATERIALS AND METHODS

### Animals and experiment design

This animal trial involved multiparous Landrace × Yorkshire (L × Y) crossbred sows of similar parity and genetic backgrounds. The sows were raised and fed at the Jiade Sow Farm in Chongzuo City, China. During pregnancy, animals were fed a basal diet formulated for the gestation period, and during lactation, they were fed a basal diet designed for the lactation period. The feed formulation and its nutritional composition are provided in Table 1. The control group (*n* = 45) was fed a basal diet containing 0.27 mg Se/kg. The selenium yeast group (*n* = 43) was fed the same basal diet supplemented with selenium yeast containing 2,000 mg Se/kg. This supplementation provided an additional 0.13 mg Se/kg, resulting in a total dietary selenium concentration of 0.40 mg Se/kg from 7 days before mating to gestation day 21. During the remaining period, sows were fed the basal diet. During gestation, the daily feed allowance was divided into two equal portions (7:00 and 14:00), and sows had *ad libitum* access to water. Sows were transferred to the farrowing unit prior to parturition. During lactation, all sows were fed *ad libitum*.

**TABLE 1:** Ingredients and nutrient composition of the basal diets

Item	Basal diet (gestation)	Basal diet (lactation)
Ingredients (%)		
Corn	51	50
Soybean meal	15	9
Soybean hulls	13	0
Wheat bran	10	4
Dried beet pulp	6	2
Glucose		1
Flour	0	12.5
Expanded soybeans	0	8
Fermented soybean meal	0	4
Soybean oil	0	2.5
Fish meal	0	3
Premix^*a*^	4	0
Premix^*b*^	0	4
Total	100	100
Nutrient contents		
NE, MJ/kg	9.21	10.43
Crude protein	14	17
Calcium	0.85	0.9
Phosphorus	0.35	0.35
Lysine	0.65	1
Methionine	0.18	0.3

^*a*^ Premix was provided per kilogram of diet: iron 220 mg, copper 18 mg, zinc 120 mg, manganese 60 mg, iodine 0.4 mg, selenium 0.2 mg, chromium 0.2 mg, vitamin A 11,500 IU, vitamin D_3_ 3,500 IU, vitamin E 120 IU, vitamin B_1_ 3.4 mg, vitamin B_2_ 9.8 mg, vitamin B_6_ 5.8 mg, vitamin B_12_ 0.034 mg, niacin 30 mg, folic acid 4.8 mg, D-pantothenic acid 20 mg.

^*b*^ Premix was provided per kilogram of diet: iron 260 mg, copper 20 mg, zinc 140 mg, manganese 70 mg, iodine 0.5 mg, selenium 0.3 mg, chromium 0.2 mg, vitamin A 15,000 IU, vitamin D_3_ 4,000 IU, vitamin E 200 IU, vitamin B_1_ 4.0 mg, vitamin B_2_ 12 mg, vitamin B_6_ 8 mg, vitamin B_12_ 0.04 mg, niacin 40 mg, folic acid 5.0 mg, D-pantothenic acid 30 mg.

### Sample collection

Fecal samples were collected from sows on gestation days 14, 21, 107, and lactation days 7, 21. Sterile polyethylene gloves were worn, and the sow’s anus was gently stimulated to induce defecation. Fresh fecal material was collected from the central portion of each sample into sterile cryogenic tubes and immediately snap-frozen in liquid nitrogen. Blood samples were collected from sows on gestation days 14, 21, and 28. Blood was drawn from the anterior vena cava, followed by centrifugation at 3,000 × *g* for 15 min. The resulting serum was aliquoted into sterile cryovials and immediately snap-frozen in liquid nitrogen. All samples were subsequently transferred to a −80°C freezer for long-term storage and further analysis.

### Microbial genomic DNA extraction and 16S rRNA gene sequencing

Bacterial genomic DNA from fecal samples was extracted using the FastPure Stool DNA Isolation Kit. DNA quality was assessed using 1% agarose gel electrophoresis, and DNA concentration and purity were measured with a NanoDrop 2000 spectrophotometer (Thermo Scientific, USA). For the bacterial community, the 16S rRNA gene was amplified using the universal bacterial primers 27F (5′-AGRGTTYGATYMTGGCTCAG-3′) and 1492R (5′-RGYTACCTTGTTACGACTT-3′). Purified amplicons were pooled in equimolar amounts, and DNA libraries were constructed using the SMRTbell Prep Kit 3.0 (Pacific Biosciences, California, USA), following the manufacturer’s instructions. The purified SMRTbell libraries were sequenced on the PacBio Sequel IIe System (Pacific Biosciences, California, USA) by Majorbio Bio-Pharm Technology Co. Ltd. (Shanghai, China). High-fidelity (HiFi) reads were obtained from the subreads, generated using circular consensus sequencing via SMRT Link v11.0. HiFi reads were barcode-identified and length-filtered, retaining the 1,000–1,800 bp sequence. After quality control, a total of 11,245,594 high-quality reads were obtained from the fecal samples. The number of reads ranged from 27,283 to 61,432. The optimized-HiFi reads were clustered into operational taxonomic units (OTUs) using UPARSE 11 with a 97% sequence similarity level. To minimize the potential impact of sequencing depth on subsequent alpha and beta diversity analyses, all samples were rarefied to 9,075 sequences per sample. The taxonomy of each OTU representative sequence was analyzed by RDP Classifier version 2.2 against the 16S rRNA gene database (NT_Taxon_core_v2024/16s_bacteria) using a confidence threshold of 0.7.

Bioinformatic analyses of fecal microbiota were carried out using the Majorbio Cloud platform (https://cloud.majorbio.com). Based on the OTUs information, rarefaction curves and alpha diversity indices, including observed OTUs, Shannon index, and Chao1 richness, were calculated with Mothur v1.30.2. The similarity among the microbial communities in different samples was determined by PCoA based on Bray-Curtis dissimilarity using the Vegan v2.5-3 package. The linear discriminant analysis (LDA) effect size (LEfSe) (http://huttenhower.sph.harvard.edu/LEfSe) was performed to identify the significantly abundant taxa of bacteria among the different groups.

### Untargeted-metabolomics analysis

Metabolite profiles in feces and serum were detected using liquid chromatography-tandem mass spectrometry at Majorbio. First, 50 mg of feces and 100 µL of serum were individually extracted with 400 µL of methanol–water (4:1, vol/vol) containing 0.02 mg/mL L-2-chlorophenylalanine as the internal standard. The mass spectrometric data were collected using a Thermo UHPLC-Q Exactive HF-X Mass Spectrometer equipped with an electrospray ionization source operating in positive and negative modes. Data analysis was performed on the Majorbio Cloud Platform (https://cloud.majorbio.com). Metabolites with variable importance in projection scores >1 and *P* values <0.05 were defined as differential metabolites. Differential metabolites between the two groups were mapped to biochemical pathways through metabolic enrichment and pathway analysis using the KEGG database (http://www.genome.jp/kegg/).

### Selenium concentration analysis

The concentrations of selenium in feed or serum were determined as previously described (36). Briefly, samples were digested with a mixture of nitric and perchloric acids, and selenium concentrations were quantified using a hydride-generation atomic fluorescence spectrometer (AF-610B; Beijing Rayleigh Analytical Instrument Corporation).

### Measurement of steroid hormones

Serum progesterone and estradiol concentrations were quantified using commercially available radioimmunoassay kits (Beijing North Institute of Biotechnology, Beijing, China).

### Measurement of antioxidant-related indicators

Levels of antioxidant-related indicators in serum were measured separately. Five antioxidant-related indicators, including T-AOC, GSH-Px, SOD, MDA, and CAT, were quantified using commercial kits (Nanjing Jiancheng Bioengineering Institute, Nanjing, China).

### Quantitation of short-chain fatty acids

One gram of feces was weighed, dissolved in 1 mL of methanol, and mixed thoroughly. The homogenized solution was centrifuged at 12,000 × *g* for 10 min at 4°C, and the supernatant was collected in a new tube. The supernatant was diluted with 25% metaphosphoric acid (5:1, vol/vol) and incubated overnight at 4°C. The solution was centrifuged again at 12,000 × *g* for 10 min at 4°C. Finally, SCFAs in the supernatant were analyzed by gas chromatography according to a previously published protocol (37).

### Statistical analysis

Statistical analyses were performed using GraphPad Prism 9.0 (Media Cybernetics, USA). Measurement data are presented as mean ± standard error of the mean (SEM). Statistical significance between two groups was determined by unpaired Student’s *t*-tests or Mann–Whitney U test. *P* values <0.05 were considered statistically significant.

## Data Availability

The raw 16S rRNA gene sequencing data have been deposited in the NCBI Sequence Read Archive (SRA) under BioProject: PRJNA1373502.

## SUPPLEMENTAL MATERIAL

Fig. S1 spectrum.01303-26-s0001.tif Comparison of intestinal microbial community differences between control and selenium yeast supplemented sows on gestation day 107 and lactation days 7 and 21.

Fig. S2 spectrum.01303-26-s0002.tif OPLS-DA score plots and model overview.

ASM does not own the copyrights to Supplemental Material that may be linked to, or accessed through, an article. The authors have granted ASM a non-exclusive, world-wide license to publish the Supplemental Material files. Please contact the corresponding author directly for reuse.
